# Design and Development of a 2 × 2 Array Piezoelectric–Electromagnetic Hybrid Energy Harvester

**DOI:** 10.3390/mi13050752

**Published:** 2022-05-10

**Authors:** Bing Han, Shubin Zhang, Jianbin Liu, Yanfeng Jiang

**Affiliations:** 1 Department of Electronic Engineering, Jiangnan University, Wuxi 214122, China; 6201924083@stu.jiangnan.edu.cn (B.H.); jiangyf@jiangnan.edu.cn (Y.J.); 2 Institute of Advanced Technology, Jiangnan University, Wuxi 214122, China; 3 Postdoctoral Workstation of Zhejiang Jinzhou Group Ltd., Huzhou 313000, China; 4 Department of Electronic Engineering, Wuxi Taihu University, Wuxi 214151, China; liujb@wxu.edu.cn

**Keywords:** energy harvesting technology, piezoelectric, electromagnetic, hybrid energy harvester

## Abstract

Energy harvesting technology is regarded as a feasible solution for the continuous power supply of microelectronic devices. Efforts have been made to improve the output power of all kinds of energy harvesting devices. This paper reports a 2 × 2 array piezoelectric–electromagnetic hybrid energy harvester that achieves high power output through the combination of piezoelectric and electromagnetic conversion. The harvester included four piezoelectric–electromagnetic hybrid modules, each of which consisted of a piezoelectric sheet, a permanent magnet and a wound coil. The permanent magnet, also serving as the mass block of the cantilever beam when subjected to external stimulus, contributed to a large displacement of the vibration and generated high output power. At an acceleration of 1 g and a resonance frequency of 70.4 Hz, the measured maximum output power of the hybrid energy harvester was 66.08 mW, of which the piezoelectric and electromagnetic portions were 56.96 and 9.12 mW, respectively. Furthermore, in a charging experiment, a capacitor of 23.5 mF was charged to 11.5 V within 20 s, which demonstrates a practical application of the hybrid energy harvester for microelectronic devices.

## 1. Introduction

With the rapid development of microelectronic devices, the demand for efficient and reliable power supply is increasing. At present, most microelectronic devices use batteries for power supply. However, battery replacement or charging greatly increases the cost, and the discarding of batteries also causes environmental pollution [1,2,3]. Vibration energy harvesting technology is a reliable solution to this problem. According to different working principles, vibration energy harvesting technology can be divided into piezoelectric (PEG) [3,4,5,6,7,8,9], electromagnetic (EMG) [10,11,12], electrostatic [13,14], thermoelectric [15,16] and triboelectric (TEG) [17,18]. Among them, PEG has attracted extensive attention because of its advantages of small external influence, high energy density and easy miniaturization. The working principle of PEG is based on the piezoelectric effect. When the piezoelectric material is deformed by external excitation, the bound charge in the piezoelectric material moves, resulting in a change in the charge on the electrode coated on the surface of the piezoelectric material, so as to realize the conversion of vibration energy into electric energy. Nisanth et al. designed and optimized an aluminum nitride (AlN)-based piezoelectric vibrational energy harvester (PVEH) and compatibility with a micro-electromechanical system (MEMS) technology [6]. The optimized PVEH had a trapezoidal beam and a triangular-shaped proof mass, and it generated an output power of 0.24 µW at 158.8 Hz and 0.5 g of input vibration. Compared with an array of conventional rectangular energy harvesters, it was found that the array using the proposed structure provided 1.79 times higher power at a lower resonant frequency than the conventional array. Shearwood et al. presented a wearable device that scavenged energy from bees’ mechanical vibrations through a piezoelectric beam, and it generated an output power of 3.6 µW and a peak-to-peak voltage of 0.6 V from tethered honeybee flight [7]. However, the output power of a single piezoelectric energy harvester was limited due to the volume and material limitations. Therefore, researchers attempted to hybridize piezoelectric and other energy conversion mechanisms [19,20,21,22,23,24,25,26,27,28,29,30,31,32]. Li et al. reported a hybrid energy harvester (HEH) based on TEG and PEG from low-frequency ambient vibrations [21]. The device was capable of achieving a maximum power of 19.6 mW from the two technologies combined. In addition, hybridization of TEG and EMG has also been reported, demonstrating a broadband energy harvester via combining a nonlinear stiffening effect and multimodal energy harvesting [22,23]. Hybrids of PEG and EMG technology have also attracted a large number of researchers [24,25,26,27,28,29,30,31,32].

PEG and EMG have different output characteristics. PEG can produce high voltage and low current at low frequency, while EMG often produces low AC voltage. Therefore, the combination of PEG and EMG can form complementary outputs, that is, a high voltage and high internal resistance output by the piezoelectric mechanism and a low voltage and low internal resistance output of the electromagnetic mechanism [22]. Xu et al. developed a distributed parameter model for an HEH and fabricated a mesoscale HEH, and the peak output power was 2.93% and 142.18% higher than that of standalone PEG and EMG, respectively [25]. Wu et al. designed a novel HEH that could drive microsensors for civil engineering monitoring, and the maximum output power of the HEH was 5.19 µW [27]. Jung et al. developed design guidelines and optimization strategies based on a parametric model for hybridized energy harvesters coupling two or more distinct mechanisms, demonstrating design guidelines for an oval-shaped hybrid energy harvester consisting of PEG and EMG, achieving the time-averaged power output of 25.45 mW at 60 Hz and 0.5 g input vibration [29]. Pyo et al. designed a novel galloping piezoelectric–electromagnetic energy harvester with improved output power at a wind speed of 9 m/s, 21% higher than that of a classical one that did not have a magnet in it [30]. Li et al. replaced the mass block of the basic cantilever piezoelectric energy harvester by a magnet array and added a coil array [31]. According to the experimental results, the power density of the hybrid harvester was 686 times higher. Iqbal et al. reported a multimodal hybrid piezoelectric–electromagnetic insole energy harvester with six resonant frequencies, and the output voltage of the hybrid harvester was 30% higher than the standalone piezoelectric unit [32].

In this paper, we report a 2 × 2 array piezoelectric–electromagnetic HEH. The array HEH was assembled by four cantilever hybrid energy harvesting modules, and each module was composed of a PZT ceramic sheet, a permanent magnet and a wound coil. The magnet was not only used to generate electromagnetic energy but also acted as the mass block of the cantilever beam. A finite element model for the HEH was established to analyze the vibration characteristics. In addition, the nonideal performance gain from a single HEH to the array is discussed, and the effect of a key geometry factor—the vertical distance between the upper and lower HEHs—on the output’s performance was investigated. Finally, a potential application of the array HEH was demonstrated in an experiment charging millifarad-level capacitors.

## 2. Numerical

**Figure 1:**
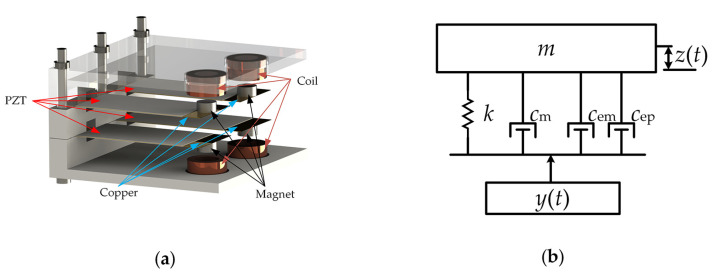
(**a**) Schematic view of the hybrid energy harvester based on PEG and EMG; (**b**) lumped model of the hybrid energy harvester.

Figure 1a shows a schematic view of the array HEH. The extremity of the piezoelectric cantilever beam was fixed on the array HEH shell to form a 2 × 2 array. Permanent magnets were arranged at another extremity, and the magnets underwent quasi-vertical motions as the device vibrated. The EMG hybridization with PEG was achieved simply by placing wound coils in the vertical direction of the permanent magnet. The wound coils were serially connected for the improved output voltage of the EMG, and the piezoelectric cantilever beams were connected in parallel to improve the output current of the PEG.

We modeled the hybrid energy harvester as a mass-spring-damper system as shown in Figure 1b. As can be seen from the figure, *c*_m_, *c*_em_ and *c*_ep_ are the mechanical, piezoelectric and electromagnetic damper constants, respectively. The vibrating structure is represented by a spring with stiffness, *k*, and mass, *m*. $y(t)=Y\sin(\omega_{s}t)$ is the source vibration function (where *Y* and $\omega_{s}$ are the source vibration amplitude and frequency), and *z*(*t*) is the relative deflection due to the source vibration. The governing equation of the relative displacement, *z*(*t*), caused by harmonic base excitation, *y*(*t*), is as follows:

(1) $$m\ddot{z}+c\dot{z}+kz=-m\ddot{y},$$

When the harvester is subjected to base excitation, *y*(*t*), the amplitude, *Z*, of the relative motion, *z*(*t*), can be written as:

(2) $$Z=\frac{r^{2}Y}{\sqrt{{\left(1-r^{2}\right)}^{2}+{\left(2\zeta_{t}r\right)}^{2}}},$$

where *r* ($r=\omega_{s}/\omega_{n}$) denotes the frequency ratio; $\zeta_{t}$ denotes the total damping ratio ($\zeta_{t}=\zeta_{m}+\zeta_{em}+\zeta_{ep}$); $\zeta_{m}=c_{m}/2\sqrt{mk}$ and $\zeta_{ei}=c_{ei}/2\sqrt{mk}$ are the mechanical and electrical damping ratios of each energy harvester in the system; $\omega_{n}$ is the undamped natural frequency of the vibrating structure.

The power output from the hybrid device can be written as:

(3) $$P_{\mathrm{hybrid}}=\frac{mr^{3}\omega_{s}{}^{3}\left(\zeta_{\mathrm{em}}+\zeta_{\mathrm{ep}}\right)Y^{2}}{{\left[\left(1-r^{2}\right)+2\zeta_{t}r\right]}^{2}},$$

When in resonance ($\omega_{s}=\omega_{n}$, r=1), the power output of the device can be simplified as:

(4) $$P_{\mathrm{hybrid}}=\frac{m\omega_{s}{}^{3}\left(\zeta_{\mathrm{em}}+\zeta_{\mathrm{ep}}\right)Y^{2}}{4\zeta_{t}{}^{2}},$$

It can be seen from Equation (4) that the output power depends on the effective mass, resonance frequency, external excitation amplitude and system damping. The power of the harvester can be improved significantly upon reasonable design.

The power generated from the standalone piezoelectric harvester, *P*_p_, irrespective of the geometry, can be written as:

(5) $$P_{p}=\frac{V_{o}^{2}R_{\mathrm{Lp}}}{{\left(R_{s}+R_{\mathrm{Lp}}\right)}^{2}},$$

where *V*_o_ is the open-circuit voltage of the piezoelectric energy harvester; *R*_LP_ is the load impedance; *R*_s_ is the source impedance. When *R*_Lp_ = *R*_s_ (impedance matching), the maximum power output can be obtained. Then Equation (5) can be simplified to:

(6) $$P_{p}=\frac{V_{o}^{2}}{4R_{s}},$$

In simulation and experiment, the piezoelectric power output, *P*_p_, equals (*V*_Lp_)^2^/*R*_Lp_.

For the peak power output of the electromagnetic technology, the load resistance, *R*_Lem_, must be matched with the coil resistance, *R*_Coil_. The maximum power output from the electromagnetic portion at resonance is:

(7) $$P_{\mathrm{em}}=\frac{{\left(NBlY\right)}^{2}}{16\zeta_{t}{}^{2}R_{\mathrm{Coil}}},$$

where *N*, *l* and *B* are the number of turns, length of the coil and the magnetic flux density.

## 3. Results and Discussion

### 3.1. Simulation

**Figure 2:**
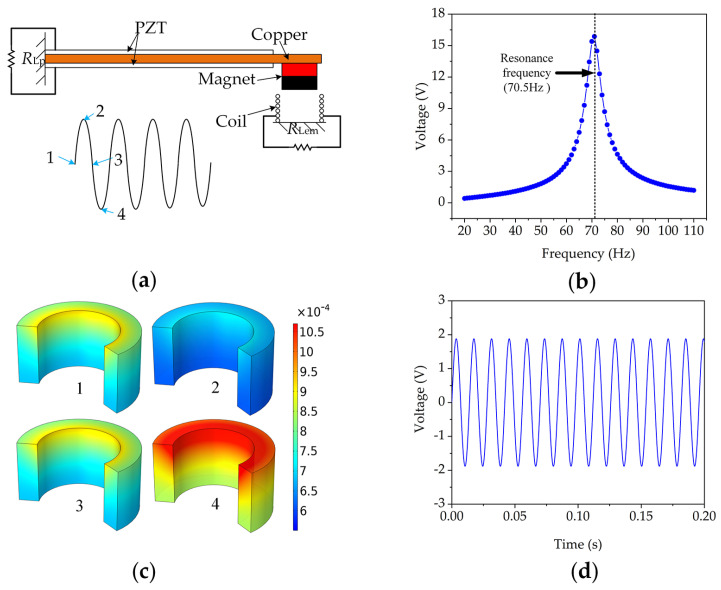
Simulation demonstration and result of a typical HEH: (**a**) schematic diagram and motion curve of a typical HEH; (**b**) open-circuit voltage of the PEG portion according to the input vibration frequency; (**c**) magnetic flux density (unit: T) in the coil at point 1, 2, 3 and 4; (**d**) open-circuit voltage of the EMG portion at 70.5 Hz.

In order to determine the approximate range of resonant frequency and output voltage, we established the model of the hybrid energy harvester using COMSOL Multiphysics 5.5 (Stockholm, Sweden). A finite element method was used to perform the characteristic analysis. The model comprised a bimorph piezoelectric cantilever beam with a permanent magnet as the tip mass and a wound coil attached on the frame; the coil and magnet were axially aligned as shown in Figure 2a. The piezoelectric beam was fixed on the base, and its vibration was harmonic. The piezoceramic operates in the d_31_ mode. It was assumed that the two piezoelectric ceramic layers were perfectly combined with the copper layer and that the conductive electrodes completely covered the top and bottom surfaces of the piezoceramic layers.

Figure 2b shows the output voltage of the PEG portion changing with the vibration frequency. A peak open-circuit output voltage of approximately 16.1 V was obtained at 70.5 Hz and a 1 g input vibration. When the HEH was subjected to external harmonic oscillation, the vibration of the permanent magnet could be regarded as a sinusoidal curve as shown in Figure 2a. Points 1 and 3 refer to the static positions of the permanent magnet, and points 2 and 4 refer to the highest and lowest positions in the vibration process of the permanent magnet, respectively. Accordingly, Figure 2c demonstrates the magnetic flux density of the coil at the four positions. Figure 2d shows a peak-to-peak value of 3.78 V for the output voltage of the EMG portion under the same stimulus condition.

### 3.2. Experimental Results

**Figure 3:**
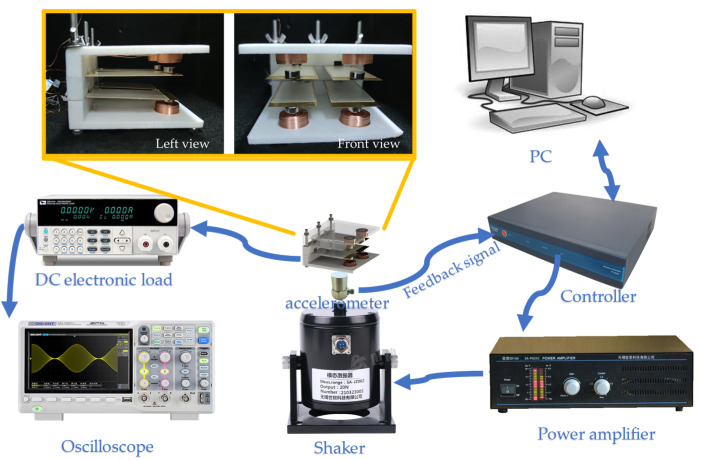
Images of the 2 × 2 array piezoelectric–electromagnetic hybrid energy harvester.

The piezoelectric–electromagnetic HEH array fabricated in this work is shown in Figure 3 as well as the experimental setup. The main instruments used in the study were a mechanical shaker (SHIAO SA-JZ002, Wuxi, China), an accelerometer (SHIAO SAPC0004, Wuxi, China), a power amplifier (SHIAO SA-PA010, Wuxi, China), a controller (ECON VT-9002, Suzhou, China) and an oscilloscope (SIGLENT SDS1104X-C, Shenzhen, China). The shell was fabricated by 3D printing, and the device’s dimensions were 90 × 90 × 55 mm. The piezoelectric sheet consisted of three layers: the top and bottom layers were piezoelectric ceramic material (PZT) of the dimensions 70 × 30 × 0.2 mm; the middle layer was made of beryllium copper with a size of 80 × 33 × 0.2 mm. One end of the piezoelectric sheet was fixed onto the shell to form a cantilever beam with long screws. When externally excited, the other end could vibrate freely. Four piezoelectric cantilever beams formed an array structure, the spacing between the upper and lower cantilever beams was 10 mm in the first place, and the distance between the left and right cantilever beams was 15 mm. A permanent magnet with a magnetization intensity of approximately 1.0 T was fixed on the other end of the copper, and it was distributed concentrically with a coil with a wire diameter of 0.2 mm and a number of 1080 turns. The permanent magnets in the vertical direction were repulsive. We connected the piezoelectric sheets in parallel and the coils in series to obtain a higher output current and voltage.

#### 3.2.1. The Performance of a Single HEH Module

**Figure 4:**
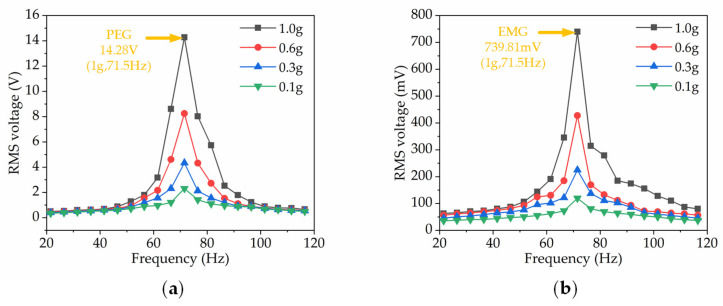
Frequency response of the PEG and EMG of a typical HEH: (**a**) PEG; (**b**) EMG.

Firstly, we examined the output characteristics of a single HEH module. The typical HEH went through a frequency sweep (20–115 Hz) under the excitation of a sinusoidal input. Notably, Figure 4a shows the output voltage of the PEG and EMG portion of the single HEH for various acceleration levels. The device resonated at 71.5 Hz, and under 1.0 g basic acceleration, the PEG produced an open-circuit voltage with a root mean square (RMS) value of 14.28 V, and the EMG produced an RMS open-circuit voltage of 739.81 mV as illustrated in Figure 4b. Compared with EMG, PEG has a wider working frequency and can achieve an output voltage of more than 4 V in a bandwidth of 20 Hz.

**Figure 5:**
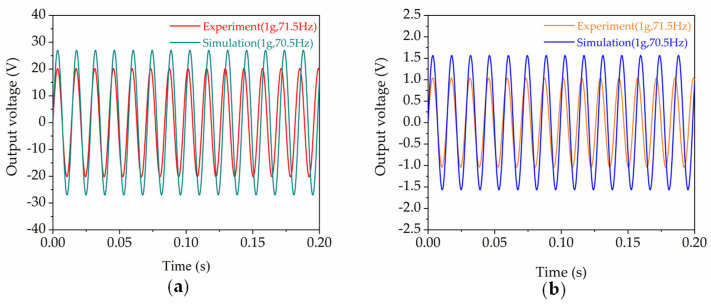
Comparison of a time-domain diagram of the experimental and simulation results of the output voltage of one HEH module: (**a**) PEG; (**b**) EMG.

Figure 5 shows a comparison between the experimental and the numerical results of the output voltage waves of one HEH module. It can be seen that the open-circuit output voltage obtained by the experiment was lower in contrast to the simulation results. In the simulation, we ignored the influence of air damping and the deformation of the copper sheet due to the prolonged vibration. This was the reason why the single HEH experiment result was lower than the simulation result; nevertheless, the deviation between the experiment and the simulation was within an acceptable range.

#### 3.2.2. The Output Performance of the Array HEH

**Figure 6:**
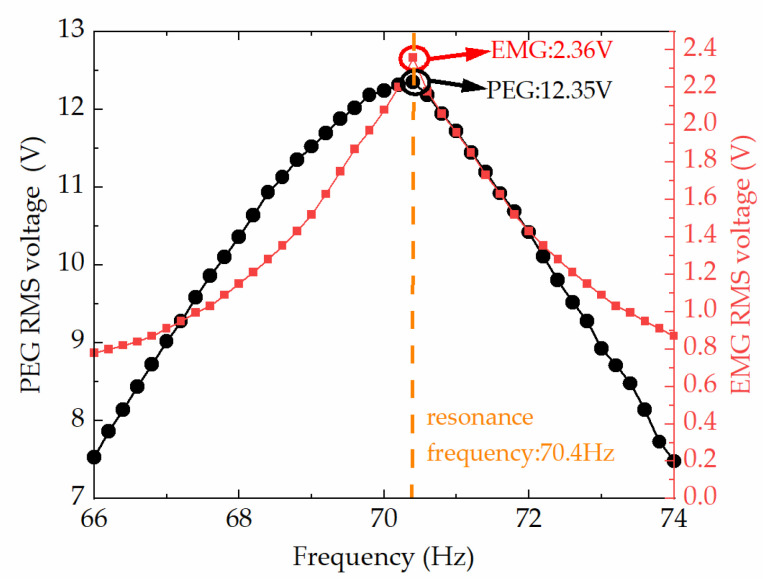
Frequency response of the array HEH.

For the array HEH, the test of frequency sweep was repeated several times to confirm the resonant frequency. The frequency sweep range was 66–74 Hz, while the acceleration was set constant at 1 g. It should be noted that the piezoelectric sheets were connected in parallel and the coils were in series for a higher PEG current and EMG voltage. As shown in Figure 6, the maximum RMS open-circuit voltage of the PEG and EMG was obtained at a resonant frequency of 70.4 Hz. The maximum RMS voltages were 12.35 V for PEG and 2.36 V for EMG, respectively. 

Compared with the one HEH module, the performance boost of the array HEH was nonideal in that it was less than four times of that of a single HEH module. This can be explained by several reasons. First of all, the intrinsic resonant frequencies differed from each HEH module due to the fact of a fabrication error. This mechanism made the individual HEHs vibrate at a frequency that was not accurately equal to the resonant frequency; therefore, not all HEHs performed at the best. However, the HEH array showed an expanded bandwidth for energy harvesting, which may also be useful because the environmental vibration energy also has a frequency range. One of the other reasons was the nonrigid property of the HEH shell to absorb some vibration energy. In addition, what interested us most was that the interaction between the permanent magnets became so strong when the vertical distance was reduced. Another damping factor comes into the picture when the vertical distance is relatively small and the movement of the magnets are one-side favored; thus, the motions can no longer be regarded as ideally sinusoidal. As the interactions between magnets were non-negligible and the vibration amplitude of the piezoelectric cantilever changed, the overall output of the array HEH was affected. 

**Figure 7:**
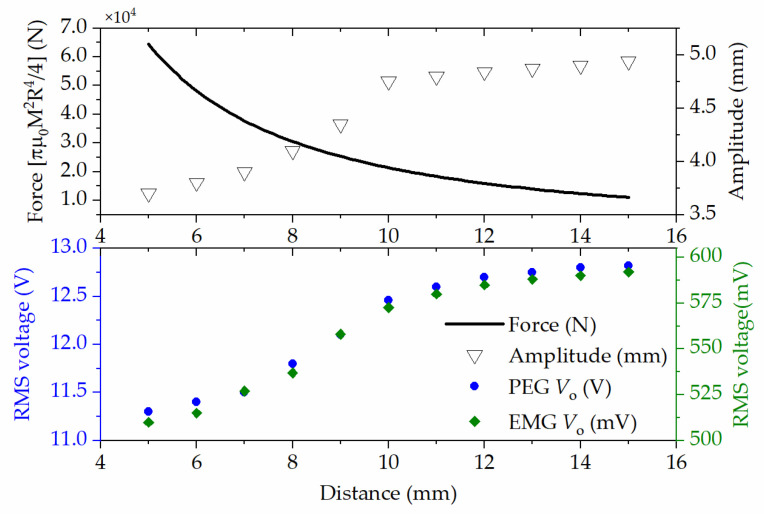
The effect of the vertical distance between magnets on the interaction, vibration amplitude and the PEG and EMG output voltages.

It can be seen from Equations (2) and (3) that the system’s total damping ratio, *ζ*_t_, was inversely proportional to the vibration amplitude and output power. Under identical vibration conditions, an enhanced damping ratio leads to a smaller vibration amplitude and lower output power. The upper and lower magnets with reduced space and opposite polarization induces stronger repulsion during vibration, which plays an additional spring element in the device. Therefore, the total damping ratio increases, and the vibration amplitude decreases. According to the Gilbert model, the force between two cylindrical magnets is approximately (radius (*R*) and length (*L*)) the following [33]:

(8) $$F\left(x\right)≃\frac{\pi \mu_{0}}{4}M^{2}R^{4}\left[\frac{1}{x^{2}}+\frac{1}{{\left(x+2L\right)}^{2}}+\frac{2}{{\left(x+L\right)}^{2}}\right],$$

where *μ*_0_ represents the vacuum permeability; *M* indicates the magnetization of the magnets; *x* denotes the vertical distance between magnets, which was herein seen as a key geometry factor for this HEH array. Figure 7 plots the force, *F*(*x*), between two magnets according to the distance, *x*. The vertical distance between magnets was set in the range of 5–15 mm. With the increase in the distance, the vibration amplitude was less affected. The figure also shows that the vertical distance had an effect on the RMS value of the open-circuit voltage. In addition, the increasing trend in the output voltage of the HEH array with a distance over 10 mm was not as obvious as when it was less than 10 mm. Thus, a column height of 8–12 mm was chosen for designing the array HEH.

**Figure 8:**
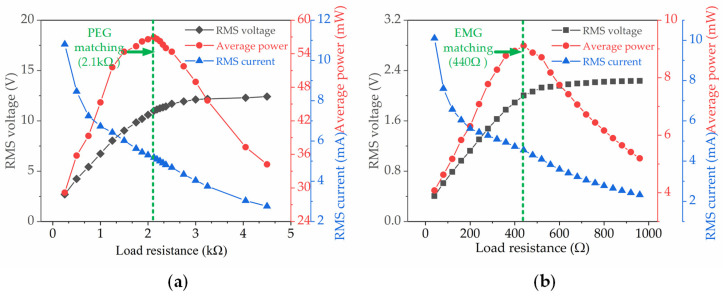
The output voltage, current and power across varied load resistances: (**a**) PEG; (**b**) EMG.

Figure 8 shows the variation in the average power (*P*_ave_), the RMS voltages (*V*_rms_) and the RMS currents of the array HEH with different external resistances. The RMS voltage was obtained from the oscilloscope. The average power was then calculated as *P*_ave_ = (*V*_rms_)^2^/*R*_L_. Figure 8a shows that the PEG of the array HEH generated an RMS voltage of 11.04 V and an RMS current of 5.16 mA when the matching impedance was 2.1 kΩ. The average power generated under this impedance reached 56.96 mW. We also measured the peak average power of the EMG when the load resistance changed. As shown in Figure 8b, the impedance matching result clearly shows that the peak average power (9.12 mW) was obtained when the resistive load was 440 Ω. The peak average power of the array HEH was 66.08 mW, which is the hybrid sum of the power generated by the two conversion mechanisms.

#### 3.2.3. Charging Experiments

**Figure 9:**
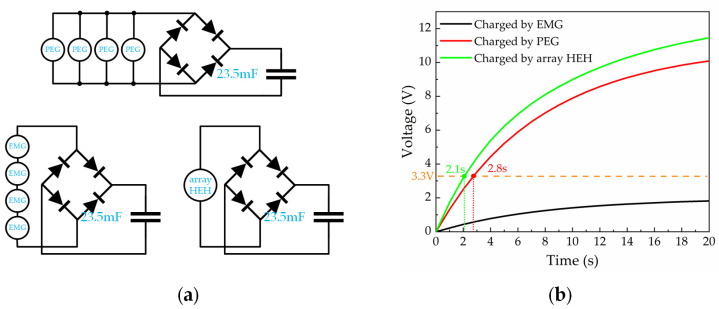
The experimental circuit and results of charging: (**a**) the circuit of the charging capacitors; (**b**) voltage curve showing a 23.5 mF capacitor charged by PEG, EMG, and array HEH.

To further verify the output performance of the array HEH, a capacitor charging experiment was conducted. Firstly, a simple charging circuit was designed and fabricated that contained the energy harvester and a bridge rectifier as shown in Figure 9a. The conditions of the tests were the same: a constant acceleration of 1.0 g, a constant frequency of 70.4 Hz and a charging time of 20 s. The PEG modules were kept in parallel and the EMG modules were arranged in series. The results are shown in Figure 9b. When the capacitor was charged with PEG or EMG alone, the voltage rose to approximately 10.1 and 1.85 V, respectively. With the hybrid of both the PEG and the EMG, the array HEH could charge the 23.5 mF capacitor to 11.5 V within 20 s. Aiming at a typical application with 3.3 V supply, the PEG charged the capacitor to 3.3 V in 2.8 s, while the array HEH took 2.1 s. Compared to PEG or EMG alone, the array HEH successfully increased the voltage of the capacitor, exhibiting an excellent charging performance and a promising application potential.

## 4. Conclusions

An array piezoelectric–electromagnetic hybrid energy harvester was designed and fabricated in this paper. The coupling of the piezoelectric and the electromagnetic energy was achieved by physically arranging the permanent magnets on the cantilever beams, which enabled the cantilever beam to obtain a customized resonant frequency and a greater vibration amplitude under external excitations. Therefore, the output power of the piezoelectric part was significantly improved. In the numerical analysis, a finite element simulation was performed, and the resonant frequency of the vibration was determined at 70.5 Hz. In the experiments, the results were in good agreement with the simulation results. A key design factor, namely, the vertical distance between the upper and lower magnets, was optimized to obtain the maximum performance of the HEH array. Under the condition of matching resistance for both PEG and EMG, they produced 56.96 and 9.12 mW output power, respectively. A capacitor charging experiment showed that the array HEH had a good energy capture performance, exhibiting great potential for powering microelectronic devices.

## Data Availability

The data supporting the findings of this paper is available from the corresponding authors on request.
